# 

**Published:** 1842-11-12

**Authors:** 


### CONTENTS OF No. 46.

Transactions of the Pathological Society,
Dr. Pepper's Case of Medullary Sarcoma of the Liver perforating the Peritoneum, - - - - - - - - 721
Dr. Pepper's Case of Cancer of the Stomach and Pancreas, - 723
Bibliographical Notices: Lectures on the Theory and Practice of Physic. By William Stokes, M. D. and John Bell, M. D. 724
Quarterly Summary of the Transactions of the College of Physicians for August, September and October, 1842, - 725
Analecta: Dr. Shortridge's Case of Empyema terminating favourably by spontaneous opening, - - - - - - - - 730
Dr. Rhind on a New Remedy for Scalds and Burns, - - 732
M. Virey's Refutation of Liebig's Theory of the uses of the Respiration and of Food, - - - - - - - - 734
&c. &c. &c.

